# Immunization Status against Measles of Health-Care Workers Operating at Three Sicilian University Hospitals: An Observational Study

**DOI:** 10.3390/vaccines7040175

**Published:** 2019-11-03

**Authors:** Claudio Costantino, Caterina Ledda, Cristina Genovese, Eleonora Contrino, Ermanno Vitale, Carmelo Massimo Maida, Raffaele Squeri, Francesco Vitale, Venerando Rapisarda

**Affiliations:** 1 Department of Health Promotion Sciences, Maternal and Infant Care, Internal Medicine and Medical Specialties (PROMISE), University of Palermo, 90127 Palermo, Italy; claudio.costantino01@unipa.it (C.C.); eleonora.contrino@unipa.it (E.C.); carmelo.maida@unipa.it (C.M.M.); francesco.vitale@unipa.it (F.V.); 2 Occupational Medicine, Department of Clinical and Experimental Medicine, University of Catania, 95123 Catania, Italy; ermanno.vitale@gmail.com (E.V.); vrapisarda@unict.it (V.R.); 3 Department of Biomedical Sciences, Dental Sciences and Morpho-functional images, University of Messina, 98125 Messina, Italy; cristinagenovese86@gmail.com (C.G.); raffaele.squeri@unime.it (R.S.)

**Keywords:** health-care personnel, vaccine-preventable disease, vaccine hesitancy

## Abstract

Measles is a highly contagious vaccine-preventable disease (VPD) that also commonly affects health-care workers (HCWs). Measles immunization of HCWs was strongly recommended by international health authorities, in order to limit the spreading of the illness to susceptible patients and colleagues. An observational study, evaluating the immunization and vaccination status against measles of HCWs working at three Sicilian university hospitals, was conducted. All subjects not completely immune (vaccinated with only one dose in their lifetime), not immune (not vaccinated or not naturally immunized), and with an unknown immunization status were considered not immunized. Among HCWs operating in the three Sicilian university hospitals, 54.6% were not immune against measles. The average age of not immune HCWs was 51.3 (SD ± 9.8), ranging between 25 and 71 years old. In particular, 46.9% of HCWs not immunized worked in “at-risk” hospital units, based on medical conditions of patients which increases the probability of contracting an infection. Vaccination coverage rates observed against measles are considerably lower than other European countries and inadequate. It is therefore crucial to tackle vaccine hesitancy among HCWs, promoting strategies targeted to evaluate immunization status against VPDs and to significantly increase vaccine coverages, such as tailored training and vaccination offer or compulsory vaccination programs.

## 1. Introduction

Measles is a highly contagious vaccine-preventable disease (VPD) with major epidemics occurring around every 2–3 years [1]. Even though a safe and cost-effective vaccine is available, 110,000 measles deaths globally were reported in 2017, mostly between children under the age of five years [1,2].

Since January 2016, a measles epidemic has been ongoing in all European Union countries, with over 50,000 cases reported [3].

From 1 January to 31 December 2018, 2526 measles cases were reported in Italy (incidence 42 cases per million inhabitants), of which 44% occurred in Sicily, the Italian region that also registered the highest incidence (222 cases per million) [4]. Among Italian health-care workers (HCWs), 115 cases were observed of which: 90 were not vaccinated, 12 cases were vaccinated with one single dose, and 3 cases were vaccinated with two doses. For one case, the number of doses administered was not clear and for nine cases, the vaccination status was unknown [4]. The median age of HCWs was 35 years and 51 HCWs (47%) developed at least one complication [4].

Measles immunization was strongly recommended by international and national health authorities, due to possible spreading to susceptible patients and the increasing risk of HCWs to contract the disease (2 to 19 times higher than the general population) [5,6,7,8].

In Sicily, the Regional Health Department adopted a specific legislation for prevention and control of the measles epidemic among HCWs, including the reporting of the vaccination and immunization status of health-care personnel operating in the regional area, with an active and free of charge offer of vaccination for not immune subjects [9,10].

Several surveys evaluated measles immunization and vaccination rates of Italian HCWs, bringing out a wide and always inadequate range of vaccination coverage [11,12,13,14].

This study investigates immunization and vaccination status against measles in Sicilian HCWs, operating in the three Sicilian university hospitals of Catania (UHC), Messina (UHM), and Palermo (UHP), in order to plan future vaccination strategies, especially among personnel working in hospital units at higher risk for contracting and transmitting measles infection.

## 2. Materials and Methods

### 2.1. Data Collection

A survey was conducted between May and July 2018 through a questionnaire, administered to HCWs working in UHC, UHM, and UHP, that accounts for 1748, 1789, and 1755 employees, respectively. Palermo, Catania, and Messina are the three major Sicilian cities, accounting for 663,770, 311,777, and 231,708 inhabitants, respectively, and the only cities with a university hospital in their area.

The recognition of the immunization and vaccination status against measles was based on national and regional decrees, in order to contrast the Italian measles epidemic that began in 2017 [9,10].

The questionnaire was divided into the following sections: (a) socio-demographic data: age, gender; (b) working activity data: type of health-care professional and hospital units (divided in “at-risk” and “not at risk” on medical conditions of hospitalized patients, which increases the probability of contracting an infection); (c) vaccination status and/or immunization status against measles, divided in: naturally immune, artificially immune (vaccinated with at least two doses), not completely immune (vaccinated with only one dose), not immune (not vaccinated and not naturally immunized), unknown immunization status.

Specifically, hospital units were considered “at-risk” if the majority of the patients admitted at the same hospital were affected by severe chronic medical conditions (such as cardiology, pulmonology, or intensive care units)

Three different methods for data detection were carried out: (1) at the UHC, a printed questionnaire was administered during the routine medical visit at the occupational medicine unit; (2) at the UHM, a printed questionnaire was directly administered for each hospital unit by the personnel operating in the hospital management; (3) at the UHP, an online questionnaire was administered throughout personal web page on the hospital web-site (restricted to unauthorized users), with a mandatory request that automatically blocked the access to the reserved area of wages sheets and attendances summary.

The ethical approval was obtained from the Ethics Committee of the University Hospital of Palermo (Palermo, Italy), in September 2017 (n.10/2017).

### 2.2. Statistical Analysis

All the information collected through the questionnaire was entered into an electronic database created by Excel 16.0 software (Microsoft Corporation, Redmond, USA).

The sample was stratified by gender, age group, professional profile, and hospital unit. Quantitative variables were normally distributed and summarized as means with their standard deviations. Differences in means were compared with the Student t-test for a paired sample.

Absolute and relative frequencies were obtained for qualitative data (gender, professional profile, hospital unit, vaccinations/immunization status, etc.). Data analysis was performed using STATA14^®^ software (StataCorp, Lakeway, USA).

## 3. Results

As reported in Table 1, 2913 out of 5328 HCWs (response rate 54.7%) working in the three Sicilian university hospitals responded to the questionnaire. Mean age was 48.2 (SD ± 10.2) years among HCWs of UHC, 49.5 (SD ± 10.5) years among HCWs of UHM, and 52.0 (SD ± 9.1) years among HCWs of UHP. The most represented age group was >50 years old, particularly in UHP, while HCWs aged ≤40 years old was more prevalent in the UHC (25.5%). More than 50% of respondents were females (57.4% Catania; 60.6% Messina, and 52.4% Palermo, respectively). The most represented HCWs category was constituted by nurses, health-care assistants, and midwives (60.2% UHC; 57.3% UHM; 52.3% UHP), followed by medical doctors (32.6%UHC; 35.5% UHM; 34.3% UHP), and other health-care technicians. Almost half of the sample worked in “at-risk” hospital units (58.1% in UHC; 41.8 % UHP; 51.5% UHM).

In Table 2, self-reported immunization and vaccination status against measles is reported. More than half of the HCWs working at the UHC was naturally immune against measles (56.6%), in contrast with data reported at the UHP (39.8%) and UHM (16.2%).

On the other hand, the higher prevalence of vaccinated HCWs was observed at UHM (17% that received at least two vaccination doses).

Finally, among HCWs operating at the UHP, 17.5% were not completely immunized and 28% did not know or did not remember their immunization/vaccination status. Overall, over 35% of HCWs of Catania and Palermo and 65% of Messina were not immune or not completely immune (received only one vaccination dose) against measles.

Specifically, as shown in Table 3, 1591 out 2913 (54.6%) HCWs operating in the three Sicilian university hospitals were not immune against measles.

The average age of not immune HCWs was 51.3 (SD ± 9.8), ranging between 25 and 71 years old. HCWs not immune at UHC (mean age 42.3 years, SD ± 10.1) were younger than UHM and UHP. The most represented age group of not immune HCWs against measles, in the Sicilian university hospitals, was ≥51 years old (56.4%), followed by 41–50 years old (30.1%).

Among not immune respondents, 59.8% were nurses, health-care assistants, and midwives, 29% medical doctors, and 11.2% other health-care technicians.

Overall 46.9% of HCWs worked in “at-risk” hospital units, with a wide range between UHC (52.6%) and UHP (50%), compared to UHM (40.1%) (see Table 3).

## 4. Discussion

Measles is a highly contagious, acute illness that could be transmitted to 75−90% of susceptible individuals in nearby places, such as health-care facilities [15].

Measles outbreaks among HCWs play a significant role in its transmission and generation of several cases with severe morbidity and mortality, high correlated costs, still in countries with long-lasting two-dose vaccination schedule [16,17].

The present survey highlighted an overall natural immunity rate against measles of 37.5% that, similarly to other developed countries, was observed in particular among HCWs ≥40 years old. In Italy, measles vaccination has been recommended at infancy since 1979. Since 1999, a circular of the Health Department recommended a trivalent vaccine: measles-mumps-rubella (MMR), for the control and elimination of these vaccine preventable diseases [18]. Despite these public health measures, a complete eradication of measles in Italy is far-distant. During the last two decades, measles vaccination coverages among children of 24 months and six years of age remain considerably lower than 95% recommended [19]. Also, among Italian HCWs, vaccination coverage against measles, mumps, and rubella remain very low [20].

Immunization rates against measles observed among HCWs operating in the three Sicilian university hospitals are totally inadequate in terms of preventing not only disease transmission by susceptible HCWs, but also nosocomial outbreaks.

In comparison with other European countries, data on measles vaccination adherence in Italy are lower. A study carried out among Austrian resident pediatricians showed high self-reported immunization rates against measles (99.2%) [21].

Moreover, a survey conducted among general physicians of the Loire Region (France), 64% of respondents stated to be immunized against measles [22]. On the other hand, Greek HCWs showed lower vaccination rate against measles (33%) [23].

In Italy, vaccination coverage for measles was indirectly associated with age, with younger HCWs that was vaccinated more than older, due to universal vaccination strategies started from 1990 and targeted to infant and adolescents [18].

In Italy it has been estimated that HCWs accounted for 7% of overall notified cases in Italy in the measles epidemic that occurred between 2017 and 2018 [24].

Regarding the epidemiological evaluation of measles epidemics that occurred in the three Sicilian UH analyzed, despite the measles circulation in Sicily in 2018 being the highest among Italian regions and one of the higher among European countries with 222.4 cases per 100,000, only one case of measles was notified, at the University Hospital of Messina, in a young medical resident [4,8].

Furthermore, during the current measles epidemic involving Europe, due to the not protective vaccination coverage among HCWs, health-care-associated transmission accounted for a critical proportion of the measles-associated burden, that also affect young HCWs [4,25,26].

Measles nosocomial infections are of particular concern in hospital units where there are patients at high risk of complications or severe diseases, such as immunocompromised patients and pregnant women [27]. Moreover, HCWs could be easily infected with a vaccine-preventable disease and could also transmit the infection to colleagues and vulnerable patients, particularly during the incubation or prodromal phase periods [17].

For all these reasons, the assessment of the immunological status of HCWs appears preeminent for preventive and occupational medicines [28].

Misdiagnosis of measles cases upon entering in emergency department is almost always a common denominator of several measles nosocomial outbreaks, making it difficult to contrast the infection spread with standard control measures (such as contact tracing, isolation, or post-exposure prophylaxis with measles vaccination) [8,16,29].

During the last decades, several factors have contributed to a decline in vaccination confidence, including the skepticism related to the effectiveness and safety of MMR vaccine [30,31].

Communication and education of HCWs regarding measles infection, measles vaccination, and barriers to vaccination are fundamental issues to achieve high vaccine uptake among HCWs and promote measles vaccination among patients [32]. In light of the recent outbreaks, a review of vaccination strategies in Italy against measles, including a mandatory vaccine administration for not immunized HCWs, should be reconsidered [33].

In Europe, only 17 out of 30 countries strongly recommend measles immunization for all HCWs, including Finland where measles vaccination is mandatory [34,35].

Some limitations affect the present study. Firstly, a lack of representativeness of our sample should be taken into account. Apart from the UH of Palermo, response rates of the Catania and Messina UHs are considerably lower than 50% of HCWs and this could not exclude a major representative of participants with positive attitudes towards measles vaccine. However, observing the consistency between results and vaccination coverage rates with different studies conducted in Italy and Europe, this limitation could only marginally affect the results of the study [12,15,21].

Moreover, the questionnaire was self-administered and it may suffer from recall bias. In particular, the evaluation of the not-immune HCWs against measles (not vaccinated/not naturally infected), in the absence of the laboratory immunological evaluation, can be altered by the fact that the infection may have been asymptomatic or the disease dates back to many years before and may not be remembered. To limit this, in all three university hospitals, concerted campaigns were organized between preventive and occupational medicine services, to recall all HCWs in order to evaluate the immunization status against measles and actively offer measles vaccination to those not immune.

## 5. Conclusions

According to data reported, immunization status against HCWs operating in the three university hospitals of Sicily, is consistently lower than recommended to limit nosocomial transmission of the disease. Vaccination strategies adopted in Italy from 1999 to date, prevalently at infancy and adolescence, did not concern the large majority of HCWs actually operating in Italian health-care facilities, that are protected against measles mostly due to contracting the natural infection.

It is therefore crucial to tackle low vaccination adherence and vaccine hesitancy among HCWs, by identifying effective strategies able to evaluate immunization status and significantly increase vaccine coverage, such as specific training dedicated to HCWs, vaccination administration at the workplace or promoting compulsory vaccination programs.

Only reaching adequate vaccination coverages against measles of HCWs could limit the risk of nosocomial measles outbreaks and avoid transmission of the disease among patients.

## Figures and Tables

**Table 1:** Socio-demographics and working characteristics of Healthcare Workers involved in the evaluation of the measles immunization and vaccination status at the three Sicilian university hospitals (*n* = 2913).

*n* = 2913 on 5328 Average Response Rate = 54.7%	Catania University Hospital	Messina University Hospital	Palermo University Hospital
Employees evaluated/ Total Employees	521/1784	822/1789	1570/1755
Response Rate	29.2%	45.9%	89.4%
Mean age ± DS	48.2 ± 10.2	49.5 ± 10.5	52.0 ± 9.1
Age groups (in years), *n* (%)			
≤40	133 (25.5)	158 (19.2)	180 (11.5)
41–50	142 (27.2)	265 (32.3)	460 (29.3)
≥51	217 (41.6)	399 (48.5)	930 (59.2)
Gender, *n* (%)			
Male	222 (42.6)	324 (39.4)	747 (47.6)
Female	299 (57.4)	498 (60.6)	823 (52.4)
HCWs type, *n* (%)			
medical doctors	170 (32.6)	292 (35.5)	538 (34.3)
nurse/ midwives/ health-care assistants	314 (60.2)	471 (57.3)	821 (52.3)
health-care technicians	37 (7.1)	59 (7.2)	211 (13.4)
Hospital Unit, *n* (%)			
at risk	303 (58.1)	397 (48.3)	808 (51.5)
not at risk	218 (41.8)	425 (51.7)	762 (48.5)

**Table 2:** Immunization and vaccination status against measles of HCWs surveyed in the three Sicilian university hospitals (UHs).

*n* = 291	Catania UH (*n* = 521)	Messina UH (*n* = 822)	Palermo UH (*n* = 1570)
	*n* (%)		
Naturally Immune	295 (56.6)	133 (16.2)	624(39.8)
Artificially Immune (received at least two vaccination doses)	32 (6.1)	140 (17.0)	98 (6.2)
Not completely immunized (receive only one vaccination dose)	46 (8.9)	82 (10.0)	274 (17.5)
Not immune (not vaccinated and not naturally immune)	14 (2.7)	244 (29.7)	134 (8.5)
Not Known/Do not remember	134 (25.7)	223 (27.1)	440 (28.0)

**Table 3:** Socio-demographics and working characteristics of HCWs not naturally or artificially immune, not completely immunized (received only one dose), and that do not know or remember their vaccination/immunization status against measles at the three Sicilian UHs (*n* = 1591).

	Catania UH	Messina UH	Palermo UH	Overall
HCWs not immune/not vaccinated/not completely vaccinated against measles, *n* (%)	194 (37.2)	549 (66)	848 (54.0)	1591 (54.6)
Mean age ± DS	42.3 ± 10.1	50.8 ± 9.6	52.7 ± 9.1	51.3 ± 9.8
Age groups (in years), *n* (%)				
≤40	48 (24.6)	78 (14.2)	83 (9.8)	209 (13.1)
41–50	62 (31.8)	186 (33.9)	231 (27.2)	479 (30.1)
≥51	79 (48.5)	285 (51.9)	534 (63)	898 (56.4)
Gender, n (%)				
Male	92 (47.4)	229 (41.7)	443 (52.2)	764 (48.0)
Female	102 (52.6)	320 (58.3)	405 (47.8)	827 (52.0)
HCWs type, *n* (%)				
medical doctors	57 (29.4)	198 (36.1)	206 (24.3)	461 (29.0)
nurse/ midwives/ health-care assistants	125 (64.4)	331 (60.3)	495 (58.4)	951 (59.8)
health-care technicians	12 (6.2)	20 (3.6)	147 (17.3)	179 (11.2)
Hospital Unit, *n* (%)				
at risk	102 (52.6)	220 (40.1)	424 (50.0)	746 (46.9)
not at risk	92 (47.4)	329 (59.9)	424 (50.0)	845 (53.1)
